# A Highly Versatile Porous Core Photonic Quasicrystal Fiber Based Refractive Index Terahertz Sensor

**DOI:** 10.3390/s22093469

**Published:** 2022-05-03

**Authors:** M. S. Aruna Gandhi, Yuanfang Zhao, H. Y. Fu, Qian Li

**Affiliations:** 1 School of Electronic and Computer Engineering, Peking University, Shenzhen 518005, China; aruna@pkusz.edu.cn; 2 Tsinghua-Berkeley Shenzhen Institute (TBSI) and Tsinghua Shenzhen International Graduate School, Tsinghua University, Shenzhen 518055, China; zhaoyf19@mails.tsinghua.edu.cn (Y.Z.); hyfu@sz.tsinghua.edu.cn (H.Y.F.)

**Keywords:** photonic quasicrystal fibers, terahertz optical fibers, refractive index sensors

## Abstract

Miniaturized real-time fiber optic sensing systems with high sensing performance are in extreme demand. In this work, we propose a novel photonic quasicrystal fiber sensor in the terahertz region and test its sensing characteristics using the finite element method. The proposed simulated sensor numerically investigates the cancer-infected cells from the normal cells in the human cervix, blood, adrenal glands, and breast based on the difference in their refractive index changes. The effective refractive index of core-guided mode is due to the interaction of light between the refractive index of the fiber material and infiltrated normal and cancer cells, respectively. The proposed sensor exhibits a high birefringence of 0.03, a low dispersion of 0.35 ps/THz/cm, along with a high numerical aperture of 0.99. Besides, the sensor holds a less-effective material loss of 2.53 × 10 ${}^{-9}$ (dB/cm), a maximum power fraction of 88.10, a maximum relative sensitivity of 82.67%, and an effective mode area of 3.16 mm ${}^{2}$ . The results envisage that the proposed sensor displays high sensing performances with a rapid cancer detection mechanism.

## 1. Introduction

The World Health Organization reports that cancer has emerged as a primary cause of death worldwide for nearly 10 million deaths in 2020 [1], demanding research on the early diagnosis of cancer cells. Among all the available methods for the early diagnosis of cancer cells, terahertz spectroscopy has been successful to a greater extent [1,2,3,4,5,6,7]. In particular, the refractive-index-based terahertz photonic sensors have extended their potential applications in life sciences, electrochemistry, environmental safety, and biomedical diagnostics [2,3,4,5,6]. In this line, terahertz photonic crystal fibers (THz-PCF) with different photonic waveguides are utilized as the point of care (POC) for various diseases by identifying distinct bio-markers such as DNA, cholesterol, glucose, and so on. In a standout, THz-PCF-based sensors have unique properties such as design flexibility, large mode area, low loss, high birefringence, and dispersion compensation facilities, which are compact and suitable for chemical and biological sensing applications [2,3]. Recently, researchers have reported significant progress in employing photonic-crystal-fiber (PCF)-based sensors in the THz regime for diagnosing different biological analytes such as proteins and nucleic acids with high efficiency and selectivity [8,9,10]. In the last few decades, several PCF structures have been designed for sensing applications, where the cladding or core holes have been filled with targeted analytes for transmission investigations [11]. Traditionally, the PCF guiding mechanisms have been differentiated into two categories, i.e., total internal reflection guidance and modified-total internal reflection guidance [12]. To achieve the maximum optical and sensing properties, various regular and irregular geometrical air hole microstructures such as square, hexagonal, elliptical, octagonal, decagonal, kagome, and honeycomb structures have been introduced into the PCF geometry [1,13,14,15,16,17,18,19].

The recent advancements in engineering the PCF-based waveguides takes advantage of unique materials such as Zeonex, Topas, high-density polyethylene, Teflon, Perspex, and Picarin to develop optical devices in the THz regime [2]. In 2018, Katyba et al. numerically investigated the hollow-core photonic crystal sapphire waveguides and fabricated them through a shaped crystal growth scheme. The proposed PCF operates in broadband transmission THz pulses with low dispersion and propagation losses, which is also efficient in various extreme conditions. The theoretical and experimental values’ agreement for temperature sensing remains valid for developing remote sensing in intense environments [20]. In 2018, Islam et al. demonstrated a modified PCF-based sensing scheme to detect chemicals, which achieved 53.22% maximum sensitivity [21]. In addition, similar work from the same group has been reported for chemical sensing with a complex kagome architecture with enhanced sensitivity of 86%, taking advantage of the arrangement of aperiodic holes in the core region [22]. A hollow-core PCF with the operating frequency varying from 0.8 to 2 THz has been reported, detecting chemical analytes such as water, ethanol, and benzene, exhibiting a maximum relative sensitivity of 96.69%, 96.97%, and 97.2%, respectively [23].

A PCF with the selective filling of cladding holes realized an epsilon-near-zero (ENZ) material achieving a high birefringence to enhance the core power fraction; relative sensitivity with near-zero dispersion at a 0.75 THz frequency has also been reported [24]. In 2019, Hasan et al. reported a heptagonal PCF to detect targeted chemical samples with a relative sensitivity of 63.24% in the THz regime [25]. A rectangular hollow-core PCF for the detection of chemical analytes with a maximum sensitivity of 89% was reported by Habib et al. [26]. Analyzing four types of materials such as Teflon, silica, polymethyl methacrylate, and chalcogenide as a fiber material, Pakarzadeh et al. reported that Teflon maintains a negligible transmission loss in the frequency range of 1.55–1.85 THz [27]. Podder et al. optimized a rectangular core PCF structure to analyze the significant components of blood, which achieves a maximum relative sensitivity of 90.8% for water, 92.14% for plasma, 92.94% for white blood corpuscles, 93.72% for hemoglobin, and 94.38% for red blood corpuscles at 1.8 THz frequency [28]. In 2021, Islam et al. optimized a wheel-shaped hexagonal porous core-based PCF sensor for detecting dairy products such as camel milk and cow milk in the THz region to achieve a high sensitivity of 81.16% with 98% porosity [29]. A highly sensitive PCF THz sensor has also been reported to detect the various cancerous cells in the human body with a high relative sensitivity of 98% [30].

Most of the porous-core-based THz PCF sensors reduce the effective material loss and achieve a high numerical aperture [13,14,15,16,17,18,19]. Furthermore, recently developed PCF-based SPR sensors have an upper detection limit taking into account the refractive index of the silica glass, which remains a challenging factor in designing a biosensor for samples having higher refractive index values [31,32]. A new class of photonic quasicrystal fibers (PQFs) with an asymmetrical arrangement of air holes in the cladding facilitates unique optical properties including low confinement loss, engineered dispersion characteristics with a flat dispersion profile for a wide range of wavelengths, high dispersion values, etc. [33,34]. Compared to the conventional photonic crystal structure, the quasi-periodic photonic crystal structure constitutes more defect modes with a high optical localization effect [35], which can be adapted in the slab, array waveguides [36,37,38,39], and in fibers [33,34,39]. To overcome the limitation of a higher detection limit, we have also proposed a ten-fold PQF for the higher analyte range from 1.45 to 1.53 with a maximum sensitivity of 6000 nm/RIU in the near IR region [40]. The name “ten-fold PQF” represents the quasicrystal tilling with local and statistical rotational symmetries identified with ten-fold air holes at the cladding and as ten-fold inflation rules. Suoda et al. proposed a six-fold PQF with the trapezoidal analyte-channel-based plasmonic sensor for detecting higher refractive index samples from 1.4 to 1.58 with a maximum sensitivity of 17,000 nm/RIU [41].

In this paper, to the best of our knowledge, this is the first existent demonstration proposing the relative sensitivity with detailed optical performance investigations achieving the maximum power fraction, high relative sensitivity, a flattened dispersion, confinement, and transmission losses with the maximum numerical aperture, as well as the specific cell detection scheme. Furthermore, the proposed novel photonic quasicrystal fiber refractive index biosensor (PQF-RIBS) also offers an admissible effective mode area with high sensor resolution, making it highly suitable for refractive-index-based chemical and biological applications.

## 2. Design Methodology of the PQF-RIBS THz Sensor

In this work, we employed a novel and simple porous-core PQF-RIBS that reduces the transmission loss, thereby achieving the flattened dispersion. The purpose of utilizing a quasicrystal-based porous core in the PQF-RIBS THz sensor is to inhibit the coupling between the core–cladding guiding modes’ transmission mechanism.

### 2.1. Structure and Method

**Figure 1:**
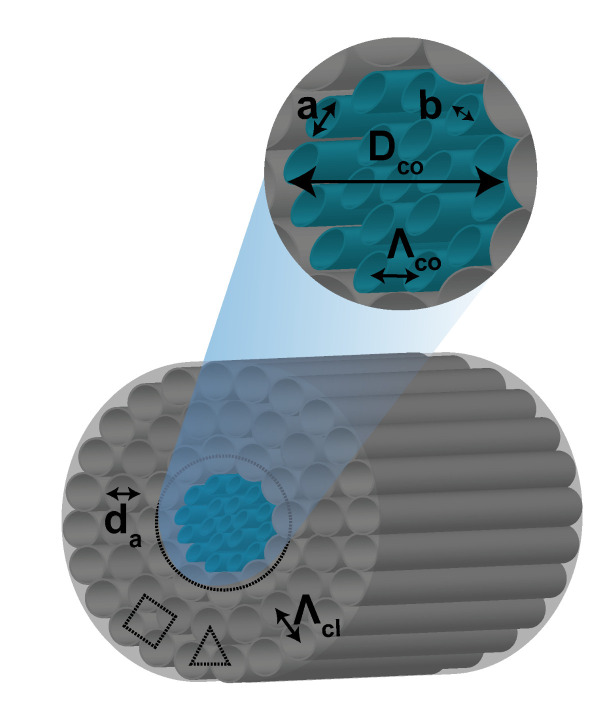
The physical architecture of the proposed PQF-RIBS in 3D.

**Figure 2:**
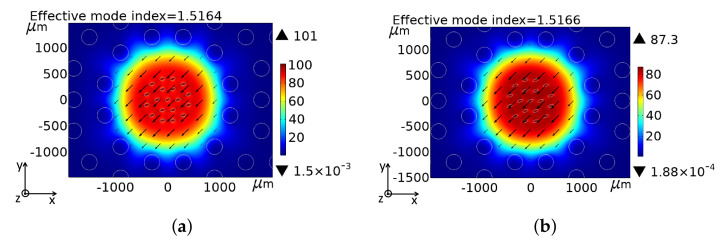
Electric field distribution of the core-guided mode of PQF-RIBS with the core, cladding, and PML focusing at the porous core region for (**a**) *x* and (**b**) *y* polarization at a 1.6 THz operating frequency. The arrows display the vectorial distribution of the corresponding transverse electric field.

The geometry of the proposed PQF-RIBS is elucidated in Figure 1, where the air holes are inserted in the cladding region made of Topas polymer. The asymmetrical air hole arrangement corresponds to the angular distribution of holes at the central point of the fiber sensor structure by the scattering radius $r_{s}=\Lambda \times \sin\left(\pi /n\right)$ where *n* represents the order of the folds and Λ describes the hole-to-hole spacing. The term “fold” is a scale of rotational symmetry. The proposed quasicrystal structure has the repetition of square and triangle arrangement of air holes in its cladding as mentioned in Figure 1. The porous core is flexible in the xy -direction, resembling a circle. The high asymmetrical rotations of air holes and optimizing the size and shape of the air hole in the core region improve the effective mode index difference within the *x* and *y* polarization direction, resulting in birefringence. In this proposed PQF-RIBS, the space between the cladding air holes and the elliptical air holes in the core is $\Lambda_{cl}$ = 600 μ m and $\Lambda_{co}$ = 250 μ m, respectively. The diameter of the cladding air hole $D_{cl}$ = 150 μ m, composed of 55 air holes. In general, the THz optical fibers including a porous core operating in the THz frequency have a core dimension of 150 to 4000 μ m [2]. In the proposed PQF-RIBS, the core region is made of thin-walled porous air holes, which are filled with a lower-refractive-index liquid than the cladding region (RI = 1.53). The normal and cancerous cells refractive indices of PC-12, Jurkat, MBA-MD-231, MCF-7, HeLA are 1.381, 1.376, 1.385, 1.387, 1.368 and 1.395, 1.39, 1.399, 1.401, 1.392, respectively, for significant cancer detection investigated in the 0.1 to 3 THz frequency band [30]. The light inside this photonic bandgap fiber is guided through inhibited coupling, where the core and cladding modes coexist. Furthermore, to ease the fabrication imperfections, our proposed PQF-based porous-core configuration can be a solution for the liquid infiltration complexity into a small air hole (350–500 μ m [2]) by offering a large internal channel (2000–2400 μ m), which is highly suitable for analyte detection. The cladding satisfies the perfectly matched layer (PML) absorption boundary condition, which helps to avoid the backscattering light from the optical fiber and maintain the field in the xy-plane, as shown in Figure 2a,b.

The significant advantages of porous-core PCFs facilitate lower material absorption loss and dispersion by engineering the structural parameters such as the diameter and pitch of the air hole and core. In addition to low loss, achieving high birefringence is a key for the applications of terahertz sensing, communications, and terahertz heterodyne detection. Highly birefringent THz fibers are designed by breaking the symmetry in the cladding and porous core. In this line, several microstructured waveguides are proposed for low loss and high birefringence in the THz band. In the proposed PQF-RIBS, the elliptical core holes’ *a* and *b* axes are 70 and 45 μ m, respectively, and their sector angle is 360° with a 20° rotation. The sector angle and rotation angle were designed by defining the ellipse structure’s size and shape with its position and rotation angle in the finite-element-scheme-based Comsol Multiphysics. Owing to the strong guiding in the THz regime and highly asymmetric structure, the effective refractive index change of the *x*- and *y*-polarized hybrid HE and EH modes results in high birefringent, achieving unique optical and sensing applications. In Figure 1, the elliptical blueish-green holes indicate the core region of the PQF-RIBS, which consists of 12 air holes. The targeted analytes are filled in the elliptical porous core for the numerical investigation. The elliptical porous holes represent the core region, and the biological cells’ refractive index is applied in the core holes.

Figure 2 describes the electric field distribution, when the electromagnetic light interacts with the chosen biological sample cells RI. In Figure 2, the arrows represent the vectorial distribution of the respective transverse electric field. It is well known that the field components of a TE wave in the xz plane (front view) are (Hx, Ey, Hz), while for a TM wave in the xz plane (front view), they are (Ex, Hy, Ez). For an incident Gaussian wave on an interface in the xy plane (front view), there is only the Ez component (out of plane), which defines this TE wave. In the PCF-based porous-core sensing approach, the porous-core holes (usually, the total diameter of the porous core is 350 μ m to 500 μ m) challenges liquid infiltration, which makes this approach very complex in terms of fabrication [2]. To ease the fabrication imperfection, our proposed PQF-based porous-core configuration is a solution for the liquid infiltration complexity into a small air hole (350–500 μ m) by offering a larger porous core (2000–2400 μ m) that is highly suitable for chemical and biological applications.

### 2.2. Two-Dimensional PQF-RIBS Simulation by FEM

The finite element method (FEM) was employed to model and characterize the sensing performances of the proposed PQF-RIBS in two-dimensional (2D) geometry using COMSOL MULTIPHYSICS—a commercially available software. In a waveguide geometry investigation, the FEM is an eminent scheme that evidences the characterization of excited core-guiding modes amid modest computer memory provisions considering mesh elements that apportion a significant geometry of the sensing scheme with relevant structural components [22,23,24,25]. In the simulation, the added physics includes the electromagnetic waves, the frequency domain interface that is available in the RF, and wave optics modules to carry out the boundary mode analysis. The perfect electric conductor was employed as the boundary condition with zero initial conditions to the exterior boundary. Based on the FEM, the optimization of mesh grids is necessary to obtain quality numerical results, as it influences the simulation accuracy. Generally, the FEM-based simulations are preferable for varying the resolution with which the field can be represented by several portions of the simulation domain.

### 2.3. Materials and Fabrication

Polymer materials such as polymethyl-methacrylate, tetrafluroethylyne, Topas, silica, and Zeonex are classed as the potential candidates to fabricate the THz sensor [2]. In this work, we considered the refractive index of the Zeonex material from the previously reported sensors [2,42]. The Zeonex material has unique optical properties such as an index of refraction of 1.53 in the THz range, which causes near to zero material dispersion, a low absorption loss of 0.2 cm ${}^{-1}$ , a higher glass transition temperature, minimal water absorption, high bio-compatibility, as well as excellent chemical resistivity, even at elevated temperatures [43]. Though Zeonex and Topas have similar optical properties, Zeonex possesses stronger chemical resistance and biocompatibility than Topas. Furthermore, Zeonex (Grade E-48R) shows a higher glass transition temperature over Topas (Grade 5013L-10) and is more appropriate for practical realization. Besides, as the material refractive index is related to effective material loss, with few Zeonex grades such as 480, 480R, and 480R, the refractive index is smaller than the Topas polymer material, which can minimize the effective material loss (check Equation (10)).

To date, various fabrication techniques have been investigated. Fabrication techniques such as stack-and-draw and sol–gel techniques are widely employed to fabricate the circular ring symmetric structured fibers [2,3], whereas extrusion and 3D printing methods have been widely suggested for asymmetrical hole structures such as rectangular and elliptical air holes [2]. The proposed PQF-RIBS can be fabricated by the traditional stack-and-draw technique, which is highly versatile and flexible. Due to the stacking of thin capillaries, various structures (i.e., hexagonal air hole lattice geometries, highly complicated superlattice geometries, are fabricated by employing the circular capillaries with the triangular, square, and elliptical holes) with different materials are successfully utilized [2]. From the literature, the fabrication of the superlattice structure is realized by employing the stack-and-draw technique to fabricate the elliptical holes and comparing with the ideal elliptical air holes results [2]. Several geometries of microstructured optical fibers with a twin core, suspended core, and either side hole were employed to fabricate the optical fiber sensing schemes for various sensing applications. Furthermore, the advanced selective filling schemes by Huang et al. [44] and Coredeiro et al. [45] that infiltrate the microstructured air holes in the core with different analyte refractive indices have already demonstrated the fabrication possibilities. Hence, the experimental realization of the proposed sensor is feasible with the existing fabrication technologies.

## 3. Theoretical Analysis of PQF-RIBS’s Characteristics

The electromagnetic (EM) mode field confinement within the PQF-RIBS is demonstrated in Figure 2a,b for the optimum model conditions at a 1.6 THz frequency for *x* and *y* polarization with the intensity scale. The electromagnetic field is strongly confined in the porous-core holes to interact with the chosen analyte RI filled in the porous core region in the THz band [22,23]. The efficiency of the strong interaction of the light and analyte RI facilitates the effective RI that can be employed to study the sensing performances of the proposed sensor, as shown in Figure 2. The PQF-RIBS efficiency is characterized by the sensing performances, which are highly dependent on the light–matter interaction. The relative sensitivity is dependent on the absorption coefficient at a frequency. The numerical outcome power-based utmost relative sensitivity of sensor *r* can be calculated as [22]

(1) $$r=\frac{n_{r}}{n_{eff}}\times F.$$

where $n_{r}$ denotes the refractive index of the chosen samples, $n_{eff}$ represents the effective index of the core-guided mode field distribution, and *F* signifies the power percentage of the guided mode and sample interaction, which is computed as [22],

(2) $$F=\frac{\int_{sample}R_{e}\left(E_{m}H_{n}-E_{n}H_{m}\right)dxdy}{\int_{total}R_{e}\left(E_{m}H_{n}-E_{n}H_{m}\right)dxdy}\times 100,$$

where $E_{m}\&E_{n}$ and $H_{m}\&H_{n}$ are the guided mode electric and magnetic field polarization, respectively. In the proposed PQF-RIBS, with the porous-core modes of the *x* and *y* polarization, the propagation constant is significantly different for each polarization. The birefringence or effective refractive index difference between different core-guided modes of the *x* and *y* polarization of the fiber can be calculated by [22]

(3) $$B=\Delta n_{xy}=\left|n_{x}-n_{y}\right|,$$

where $n_{x}$ or $n_{y}=x$ or *y* polarization of the proposed PQF-RIBS. To estimate the sensing, the output power that confines through the porous core is expressed as [46]

(4) $$P=Sin^{2}\frac{|n_{x}-n_{y}|\pi fl}{c},$$

where *l* implies the transmission length along the fiber. Due to the strong transmission efficiency of the dispersion of the fiber, to be ultra low and flat as feasible with a dramatic variation, this creates a significant efficacy variation over the bandwidth. As the material dispersion of Zeonex balances inside the examined frequency band, the waveguide dispersion of the proposed PQF-RIBS is shown by [22]

(5) $$\beta_{2}=\frac{2}{c}\frac{dn_{eff}}{d\omega }+\frac{\omega }{c}\frac{d^{2}n_{eff}}{d\omega^{2}},$$

where ω=2πf is the central angular frequency. Furthermore, the effective mode field area of the guided mode is computed by [22]

(6) $$A_{eff}=\frac{{\left(\int \int |E\right|}^{2}{dxdy)}^{2}}{\left(\int \int |E{|}^{4}dxdy\right)},$$

where |E| is the magnitude of the core-guided mode field area. The sensitivity of the sensor based on the wavelength interrogation technique can be calculated by [46]

(7) $$S_{f}\left(\mathrm{THz}\;{\mathrm{RIU}}^{-1}\right)=\frac{\Delta f_{dip}}{\Delta n_{a}},$$

where $\Delta f_{dip}$ is the shift in the frequency dip relevant to the variation in analyte refractive index $\Delta n_{a}$ of the transmitted core-guided mode. The resolution of the sensor can be calculated by

(8) $$R_{f}\left(\mathrm{RIU}\right)=\Delta n_{a}\times \frac{\Delta f_{min}}{\Delta f_{dip}},$$

where $\Delta f_{min}$ indicates the instrumental dip frequency resolution (assumed to be 0.1 THz). The refractive index is a ratio; therefore, it is dimensionless. However, in the SPR context, the refractive index unit (RIU) is used to refer to the minimum detectable range of refractive index change. Besides, the desirable numerical aperture requires a wide and high core–cladding index contrast for better sensing, which is responsible for the maximum numerical aperture, which can be expressed as [22]

(9) $$\mathrm{NA}=\frac{1}{\sqrt{\frac{1+\pi A_{eff}f^{2}}{c^{2}}}}.$$

In the THz operating range, the inevitable specific loss mechanism for PCF is the effective material loss (EML), which occurs when the EM field is absorbed by the bulk material, and it is computed by [22]

(10) $$\mathrm{EML}=\sqrt{\frac{\epsilon_{0}}{\mu_{0}}}\frac{\left(\int_{mat}n_{mat}|E{|}^{2}\alpha_{mat}dA\right)}{\;\mathrm{mod}\;\int_{all}S_{z}dA},$$

where $\epsilon_{0}$ and $\mu_{0}$ are the electric permittivity and permeability in a vacuum and $n_{mat}$ and $\alpha_{mat}$ are the refractive index of the Zeonex material and the bulk material’s absorption loss ( $n_{mat}$ = 0.2), respectively. Here, $S_{z}$ , represents the *z*-component of the Poynting vector stated as $S_{z}=\frac{1}{2}Re\left(E\times H^{*}\right)z$ , where *E* and $H^{*}$ mean the complex conjugate of the magnetic field.

## 4. Result Analysis and Discussion

To investigate the sensing performances of the proposed PQF-RIBS, the refractive indices of cancerous cells and their corresponding normal cells were taken into consideration. The effective RI change of the normal and cancerous cells facilitates the sensing performances of the proposed sensor. For this simulation, the physical nature of the cell such as the shape, size, and orientation was generally not considered. Furthermore, the proposed refractive-index-based sensor detects the change in the refractive index of the normal and cancerous cell. Therefore, the physical nature of the cell was not taken into account.

We investigated the characteristics of the proposed PQF-RIBS properties such as the relative sensitivity, dispersion, effective mode area, effective material loss, transmission analysis, and power fraction by utilizing the FEM. The refractive index of the normal and cancerous cells were infiltrated in the porous core for numerical investigations. According to the mode coupling theory, to demonstrate the process of developing the optimized PQF-RIBS filled with the targeted analyte for *x* and *y* polarization, the effective refractive index difference, the propagation constant, and $\beta_{2}$ , of the *x* and *y* polarization of the core-guided modes, were calculated. When the optical power transmits within the PQF-RIBS, the two polarization modes, i.e., *x* and *y* polarization, reveal the effective refractive index of the targeted normal and cancerous cells that were investigated for the operating frequency ranging from 0.5 to 1.5 THz. Figure 2a,b represent the enlarged scale, focusing the porous core region for *x* and *y* polarization of the fundamental core-guided mode field transmission for a targeted analyte.

**Figure 3:**
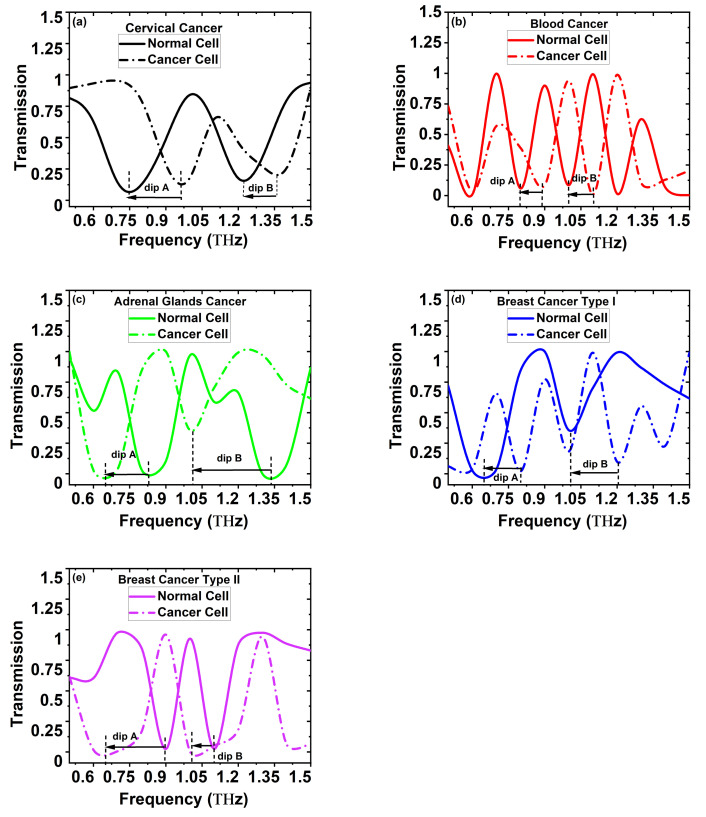
(**a**–**e**) Transmission spectrum of PQF-RIBS for *x*-polarized fundamental core-guided mode at a 1 THz operating frequency for different cancer cell types.

Next, we performed the transmission studies of normal and cancerous cell detection of different cancer cell types. The normal and cancerous cells’ refractive index indicate different transmission dip values achieved at various frequencies that are defined as the frequency dip, exhibiting different sensitivity owing to their different values of the frequency dip shift of each targeted cell type. For a transmission length of one meter, the transmission dip frequency shifts $\Delta f_{dip}$ 0.75 and 1.22 THz for the normal cervical cell and 0.96 and 1.36 THz for the cervical cancerous cell, respectively, and is shown in Figure 3a. Figure 3a–e show that the transmission spectrum’s identity is sinusoidal and that the total transmitted optical power should be denotative of the smallest value of the sinusoidal curve rather than any defined unit. Equation (6) represents the transmission spectrum of the proposed porous-core PQF-RIBS capable of cancer detection, which varies between 0 to 1 and operates in the frequency range of 0.5 to 1.5 THz. The transmission spectrum of the proposed porous-core PQF-RIBS is sinusoidal for normal and malignant cell types, as shown in Figure 3a–e. The maximum refractive index difference between the normal and cancerous cells is considered to be the detection limit ( $\Delta n_{a}$ ) of the proposed sensor. The $\Delta n_{a}$ values of PC-12, Jurkat, MBA-MD-231, and MCF-7 are matched to 0.014, and that of HeLA is 0.24. The maximum detection limit of the proposed PQF-RIBS was found to be 0.24. As the transmission spectra of normal and cancerous cells have unique refractive indices, the number of dips varies based on their effective index values.

In Figure 3a, the dip frequency shifts (dip A and dip B) between the normal and cancer cells are 0.21 and 0.14, respectively. From Equation (9), the calculated spectral sensitivity of the proposed PQF-RIBS for cervical cancer as shown in Figure 3a is 7.08 THz/RIU and 5.83 THz/RIU for dip A and dip B, respectively. Figure 3b is the detection of the dip frequency shifts for blood cancer, which are 0.10 and 0.11 THz, and the maximum sensitivity achieved is 7.50 THz/RIU and 7.85 THz/RIU for dip A and dip B, respectively. In Figure 3c, it is described that the dip frequencies shift for adrenal gland cancer 0.19 and 0.33 THz, and the spectral sensitivities achieved are 12.50 THz/RIU and 23.57 THz/RIU for dip A and dip B, respectively. In Figure 3d, it is noticed that the dip frequencies shift for breast cancer type I is 0.19 and 0.33 THz, and the maximum spectral sensitivity achieved is 18.57 THz and 13.57 THz for dip A and dip B, respectively. Figure 3e examines that the dip frequency shift for breast cancer type II is 0.15 and 0.19 THz with the highest spectral sensitivity achieved being 12.14 THz and 5.71 THz for dip A and dip B, respectively. The average spectral sensitivity of the proposed PQF-RIBS for adrenal gland cancer, blood cancer, breast cancer type I, breast cancer type II, and cervical cancer from Equation (9) are 6.45 THz/RIU, 7.67 THz/RIU, 18.03 THz/RIU, 9.96 THz/RIU, and 8.92 THz/RIU, with the spectral resolution computed from Equation (10) being 1.12 × 10 ${}^{-4}$ , 1.86 × 10 ${}^{-4}$ , 7.54 × 10 ${}^{-5}$ , 1.15 × 10 ${}^{-4}$ , and 1.98 × 10 ${}^{-4}$ , respectively. Besides, the proposed PQF-RIBS facilitates a high potential optimized sensing performance with prompt diagnoses of cancer through cell liquid regarding the normal cells. As is the case, the numerous virus-related cancers apparent with various optical parameters change their internal protein structure, and the refractive index of normal and cancerous cells is unique. The effective RI change of the normal and cancerous cells facilitates the sensing performances of the proposed sensor. Next, we also explored the basic optical properties such as birefringence, dispersion, NA, EA, and EML related to the core-guiding mode field investigations, as these are basic properties of the fiber-based sensors.

## 5. Robustness of the Proposed PQF-RIBS

Furthermore, the structural parametric analysis was performed for the robustness in fabrication tolerance by modifying the optimal specifications of the novel PQF-RIBS. The proposed PQF-RIBS can be fabricated by the traditional stack-and-draw technique. With state-of-the-art fabrication technology, the variation of ±14.3% from the optimal value is acceptable. The optimized structural parametric values are $D_{cl}$ = 150 μ m, $\Lambda_{cl}$ = 600 μ m, $\Lambda_{co}$ = 250 μ m, and the elliptical holes’ axes *a* and *b* are 70 and 45 μ m, while the sector angle is 360° with a 20° rotation.

**Figure 4:**
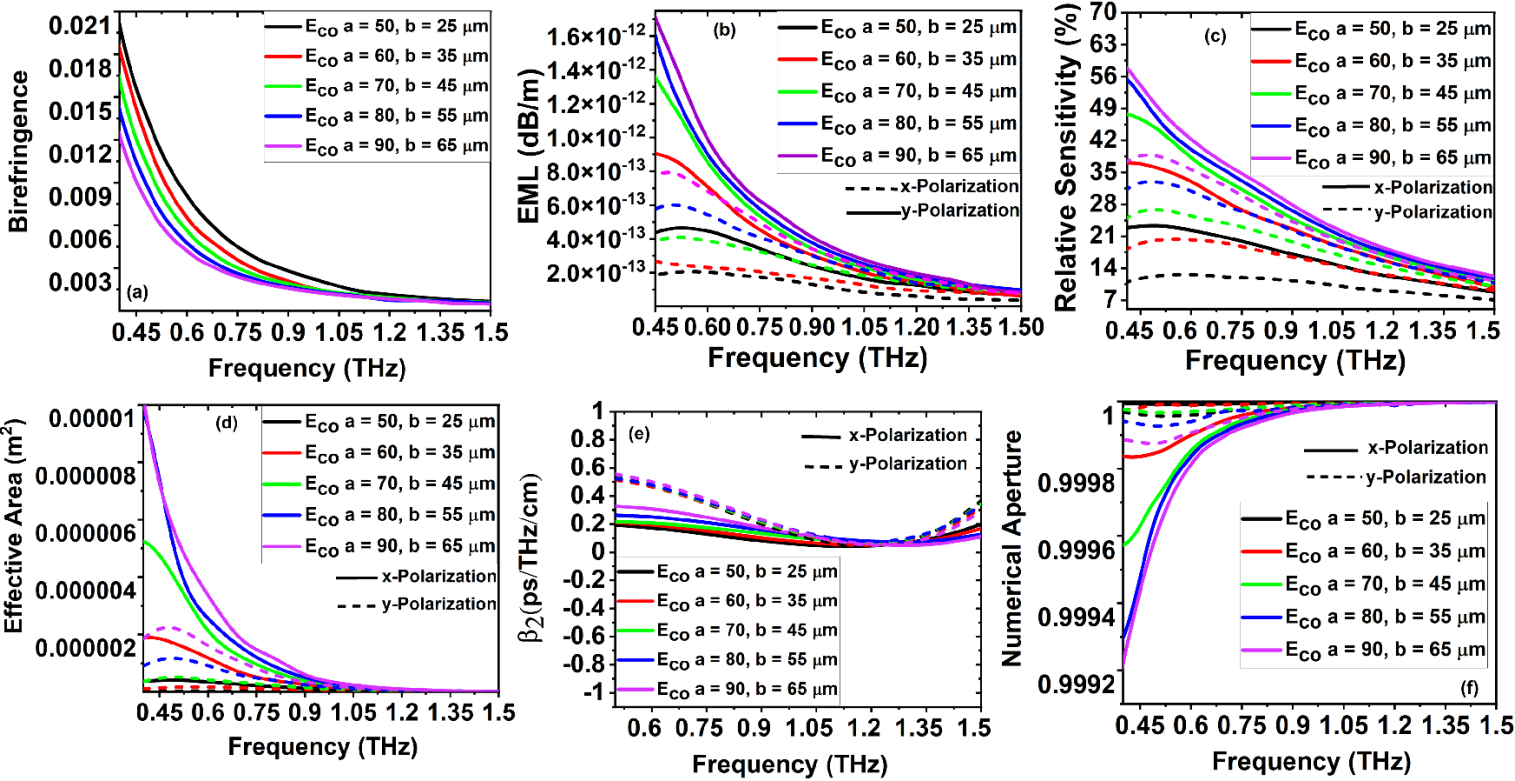
(**a**–**f**) Optimization of birefringence, effective mode loss, relative sensitivity, effective mode area, dispersion, and numerical aperture versus frequency, respectively, at the targeted analyte refractive index, n ${}_{a}$ = 1.392, for the elliptical core hole, E ${}_{co}$ , varied from *a* = 50 to 90 and *b* = 25 to 65 μ m.

Figure 4a depicts the variation in birefringence with the increase in frequency for the variation of ±14.3% from the optimized elliptical core holes of $E_{co}$ , *a* = 70 and *b* = 45 μ m, over a frequency range of 0.4 to 1.5 THz. As a result of the quasicrystal tilling in the cladding region, the *x* and *y* polarization of the core-guided effective mode values varies as a function of frequency. As the refractive index values of the core and cladding are different, the less core porousness has high birefringence, also causing low material losses, which balance between the birefringence and effective model losses. The EML of the novel PQF-RIBS as a function of frequency is shown in Figure 4b. The EML loss increases with the increases in core hole porosity. It is important to note that the EML is minimum for the high porous-core hole arrangements as the volume of material is less, causing low material loss. Figure 4c represents the relative sensitivity at various core porousness as a function of frequency. We achieved a maximum power confined at a lower porosity for *x* polarization core-guided modes than the *y* polarization mode with an increase in frequency. For the optimized core porousness and the major axis core length and frequency, the relative sensitivity with the targeted analyte for the *x* polarization is 47.85%. Low nonlinearity is necessary for long-distance terahertz transmission, which usually depends on the effective mode area. Figure 4d depicts the effective mode area of the core-guided fundamental mode with *x* and *y* polarization as a function of frequency.

For the optimized structural parameters at elliptical porousness $E_{co}$ , *a* = 70 and *b* = 45 μ m, we achieved an admissible effective mode area of 5.24 and 0.36 mm ${}^{2}$ for *x* and *y* polarization, respectively. The proposed sensor facilitates less nonlinearity owing to its higher value in the *x*-polarized mode over the *y*-polarized core-guided mode. Figure 4e describes the dispersion variation as a function of frequency for *x* and *y* polarization, respectively. The material dispersion is due to the dependency of the refractive index on a frequency that limits the bandwidth of the proposed PQF-RIBS. We calculated the waveguide dispersion using Equation (7). From Figure 4e, it is clear that the waveguide dispersion is flatter and lower for the *x* polarization core-guided mode. For the optimized porous core hole parameters, $E_{co}$ , *a* = 70 and *b* = 45 μ m, we achieved a flat dispersion of 0.32 and 0.11 ps/THz/cm in the frequency range from 0.5 to 1.5 THz for both *x* and *y* polarization, respectively. Figure 4f represents the NA as a function of frequency, where the NA increases along with the frequency. For the optimized core parameters $E_{co}$ , *a* = 70 and *b* = 45 μ m, the minimum NA achieved was 0.9995. Aimed at porous sensors, the change in the refractive index can be inspected when the targeted sample enters the pores; the effective refractive index of the sample and porous core material with the surrounding medium facilitates the optical responses of the proposed porous core sensor. From Figure 4a–f, the detailed simulation results in evidence that the core porosity of the highest diameter achieves admissible optical and optimized sensing performances, which is more promising for the structural dimensions for the practical realization.

In addition, to attain better fabrication and practical realization of the proposed PQF-RIBS, the optimization of porous-core hole pitch variation was investigated. All the structural parameters were fixed to their optimized values, and the core pitch value, $\Lambda_{co},$ was varied from 150 to 350 μ m. The investigation of porous-core hole pitch variation gave insight into the material density-of-states, which helps to achieve a small molecule diagnosis, a density factor that is required considering the optical performances derived from the pore materials. The volume of targeted samples filled in the porous and nonporous sensing medium can result in the maximum number of molecules adhered to the porous sensor. A particular density factor enhances the spectral shift in the porous-core-based biosensors.

**Figure 5:**
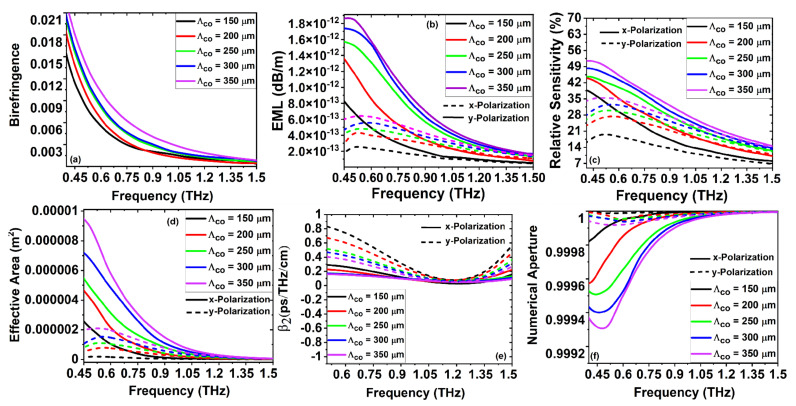
(**a**–**f**) Optimization of birefringence, effective mode loss, relative sensitivity, effective mode area, dispersion, and numerical aperture versus frequency, respectively, at the targeted analyte refractive index, n ${}_{a}$ = 1.392, n ${}_{a}$ filled elliptical core hole E ${}_{co}$ , *a* = 70 and *b* = 45 μ m, for the elliptical core pitch varied from 150 μ m to 350 μ m.

Figure 5a–f describe the optical performances of the proposed PQF-RIBS such as B, EML, RS, EA, β , and NA for both *x* and *y* polarization. From Figure 5a,c, it is clear that when $\Lambda_{co}$ is varied from 150 to 350 μ m, the birefringence increases admissibly and the fractal power decreases to reduce the relative sensitivity. Furthermore, from Figure 5b,d, it is clear that the EML and effective mode area attain the same values and the fundamental core-guided mode confinement becomes relatively strong, as expressed in the intervals of both the *x* and *y* polarization of $\Lambda_{co}$ . Figure 5e,f illustrate the adequate increase of dispersion while the values of the NA are sustained well. Besides, the diameter of the cladding of the proposed PQF-RIBS was varied to analyze the fabrication tolerance for ±6.67%. All the structural parameters were fixed to their optimized values, and $D_{cl}$ was varied for the optimization investigation. The variation of the diameter of an effective cladding can enhance the confinement within the porous-core refractive index.

**Figure 6:**
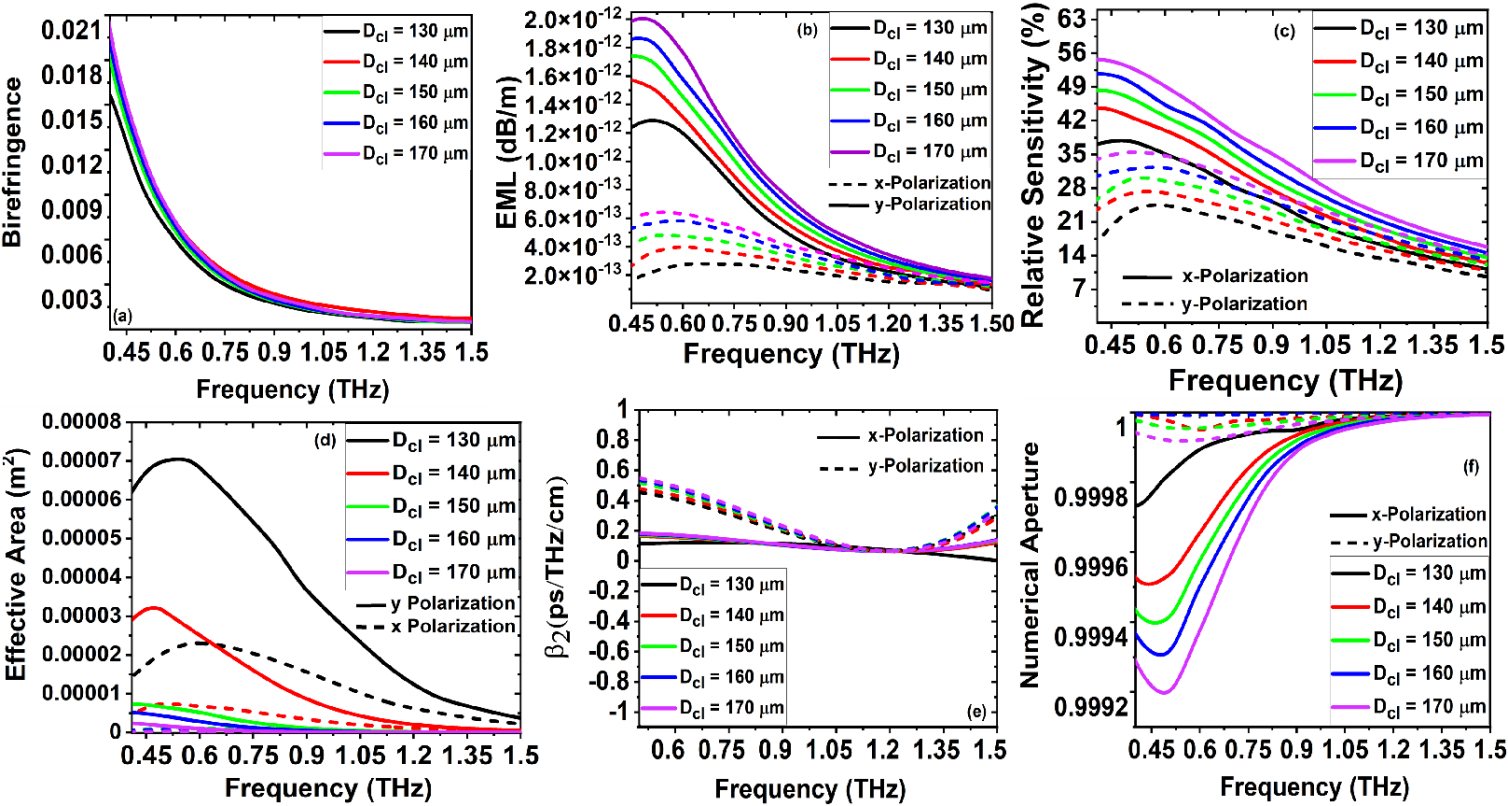
(**a**–**f**) Optimization of birefringence, effective mode loss, relative sensitivity, effective mode area, dispersion, and numerical aperture versus frequency, respectively, at the targeted analyte refractive index, n ${}_{a}$ = 1.392, for the filled elliptical core hole E ${}_{co}$ , *a* = 70 and *b* = 45 μ m, when the cladding diameter, D ${}_{cl}$ , is varied from D ${}_{cl}$ = 130 μ m to 170 μ m.

It is clear that for the proposed sensor structure, varying the porous core can effectively decrease the cross-sectional area, which can be appropriate for the targeted analyte detection. As the sensor considers filling the analytes near the cladding region, in the practical realization, the effective cladding is determined by the cladding density-of-states, chemical attraction, and accessible binding sites. Thus, the modal confinement requires the upper bound computation. Figure 6a–f describe the optical performances of the proposed PQF-RIBS such as B, EML, RS, EA, and NA at a 0.4 THz frequency for both *x* and *y* polarization. The optimization of $D_{cl}$ reduces the birefringence, thereby increasing the fractal power and increasing the relative sensitivity, as shown in Figure 6a,c. From Figure 6b,d, it is clear that the EML increases with the increase of the effective mode area for both *x* and *y* polarization. Figure 6e,f show that, by engineering $D_{cl}$ , the dispersion can be minimized for both *x* and *y* polarization. The values of the NA explain that the confinement is due to the larger density-of-states and arrangement of air holes in the cladding region, which is supported through the light–matter interaction inside the material. Over the above optimization structural parameters, the cladding pitch variation of the proposed PQF-RIBS is engineered for the fabrication and practical realization strategy. An admissible variation of ±14.3% for the optimization to perform is adequate. The chosen air holes’ pitch variation for the geometrical investigations with the modal confinement remains localized in the cladding region.

**Figure 7:**
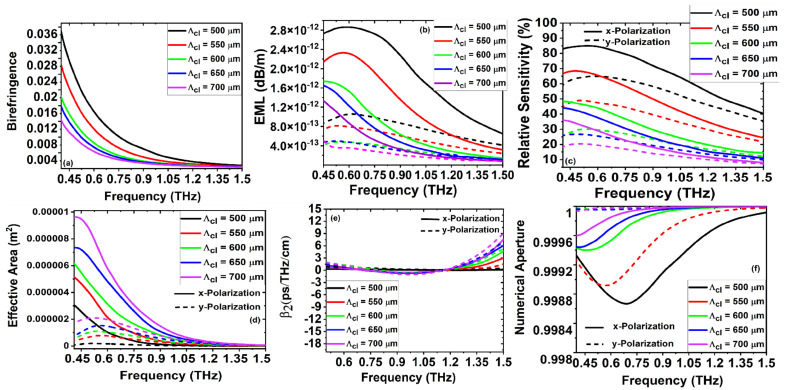
(**a**–**f**) Birefringence, effective mode loss, relative sensitivity, effective mode area, dispersion, and numerical aperture versus frequency, respectively, at the targeted analyte refractive index, n ${}_{a}$ = 1.392, for the filled elliptical core hole E ${}_{co}$ , *a* = 70 and *b* = 45 μ m, when the cladding holes’ pitch, $\Lambda_{cl}$ , is varied from 500 to 700 μ m.

Figure 7a–f describe the optical properties of B, EML, RS, EA, β , and NA for both *x* and *y* polarization, respectively, for the proposed PQF-RIBS, when the cladding pitch value is varied from 500 to 700 μ m. As $\Lambda_{cl}$ was engineered from 500 to 700 μ m, the values of birefringence as depicted in Figure 7a increase over the geometrical optimization of $E_{co}$ , $\Lambda_{co}$ , and $D_{cl}$ . As discussed in Figure 4a–f–Figure 6a–f, the relative sensitivity reaches a maximum and is shown in Figure 7c. For the larger cladding pitch value, the material around the core is high, which allows a higher fraction of light to travel and enhance the core mode area to achieve the maximum relative sensitivity, as described in Figure 7c. To ensure easier fabrication and high sensitivity, the optimum cladding pitch is defined at the point where the relative sensitivity is a maximum of 82%. Furthermore, an infinitesimal difference can enhance the relative sensitivity, and the admissible space between the core and cladding air holes helps to realize the amended fabrication. From Figure 7b,d, it is obvious that the values of EML are high compared to Figure 4a–f–Figure 6a–f; for the optimized values of $\Lambda_{cl}$ , the maximum effective mode area was observed. For the optimization of $\Lambda_{cl}$ , Figure 7e,f describe the admissible values of dispersion and the NA. From Figure 4b, Figure 5b, Figure 6b and Figure 7b, the material concentration in the core is derived from the diameter size, as well as porosity, which comprise the primary cause that leads to the high EMLs. The maximum core porosity yields less material concentration in the core area. The EML decreases with an increase in frequency, as well as when the porosity increases, the EML values also increase due to the change of the solid HRS material with low-index air in the core. As a result of the number of air holes present in the cladding, the proposed PQF-RIBS achieved a low EML of 2.53 × 10 ${}^{-9}$ (dB/cm). We proposed a new class PQF-based sensors for the first time as porous-core PQF in the THz regime with the results of admissible *B*, higher NA , lower losses, and good sensing performances over the existing sensors for bio and chemical sensing applications, as listed in Table 1.

The simulation results from Figure 4, Figure 5, Figure 6 and Figure 7 for the birefringence variation with the cladding and core air-filling fraction as a function of frequency are demonstrated. It is observed from the numerical investigations that the birefringence increases as the frequency and cladding air-filling fraction increase. By increasing the air-filling fraction, the confinement at the core increases to increase the birefringence of the proposed sensor. We proposed a new class of PQF-based sensors for the first time as porous-core PQF in the THz regime with the results of admissible B, higher NA, lower losses, and good sensing performances over the reported THz-fiber-based sensors for bio and chemical sensing applications, as listed in Table 1.

For a quasi-periodic structure, the density of the air holes in the cladding region is larger than that of the periodically arranged cladding structure. Because of the high asymmetric nature exhibited by the EML, the PQF-RIBS delivered low loss over the reported sensors [49,50,51]. A. Hassani et al. [49] reported a hexagonal arrayed porous hole microstructured optical fiber that achieved an ultra-low EML of 0.0018 cm ${}^{-1}$ . Later, S. F. Kaijage et al. [50] reported a porous-core octagonal PCF that exhibited a very low EML of 0.07 cm ${}^{-1}$ at 1 THz. Besides, Md. R. Hasan et al. [51] demonstrated a porous-core PCF that facilitated a very low EML of 0.089 cm ${}^{-1}$ at 1 THz. In 2012, B. You et al. experimentally realized the sensing with a 30 cm-long glass pipe waveguide with the hollow core filled by a low volume of different liquids with vaporizing molecules to interact with the THz waves, characterized through THz-TDS. Specifically, the volatile liquids are reactive with the plastic materials, so fabricating glass pipes is more appropriate than the polypropylene pipes for sensing vapors [52]. The small volume of liquid analyte inside the hollow core of the glass pipe waveguide can fill the hollow core with vaporized molecules to interact with the THz waves. Furthermore, the suitable fibers include the dielectric wires, ribbons, and pipes integrated for different analyte detections of liquids, solid particles, thin-films, and vapor gas-sensing applications. The volatile organic compounds included in cancer biomarkers evidence the vapor sensors for disease diagnosis. The polarity and water solubility of the volatile organic compounds are reported with their interactions with water molecules for diagnoses with vapor sensors, and a few of the volatile organic compounds’ characteristics have been reported [52]. In the proposed work, the analyte refractive index includes the concentration of the organic compounds. Consequently, the proposed sensor is more suitable for investigating the low-volume bio-liquids with vaporizing molecules passing through the fiber core. Therefore, the robustness of the structural variation validates that the proposed sensor is the most promising candidate for fabrication and experimental realization, providing more efficiency for refractive-index-based chemical and biological sensing applications.

## 6. Conclusions

In summary, we numerically investigated a photonic-quasicrystal-fiber-based refractive index sensor for significant cancer cell detection. The transmission investigation of the proposed quasicrystal-tilling-based porous-core PQF-RIBS for the refractive-index-based targeted normal and cancerous cell type analytes such as PC-12, Jurkat, MBA-MD-231, MCF-7, and HeLA evidenced the spectral sensitiveness of each analyte as 6.45 THz/RIU, 7.67 THz/RIU, 18.03 THz/RIU, 9.96 THz/RIU, and 8.92 THz/RIU with the spectral resolution 1.12 × 10 ${}^{-4}$ , 1.86 × 10 ${}^{-4}$ , 7.54 × 10 ${}^{-5}$ , 1.15 × 10 ${}^{-4}$ , and 1.98 × 10 ${}^{-4}$ , respectively. The results of enormous simulations indicated the sensor performances such as a high birefringence of 0.03, low dispersion of 0.35 ps/THz/cm, high numerical aperture of 0.99, and less effective material loss of 2.53 × 10 ${}^{-9}$ (dB/cm), with a maximum CPF of 88.10 and a maximum relative sensitivity of 82.67% for the effective mode area of 3.16 mm ${}^{2}$ . Furthermore, the optimization of the geometrical parameters for the elliptical core hole diameter and pitch and circular cladding hole diameter and pitch was investigated for the feasibility of the state-of-the-art fabrication and practical realization of the technology. The proposed sensor is a promising candidate for normal and cancerous cell detection, as well as for the refractive-index-based bio and chemical sensing applications.

## Figures and Tables

**Table 1:** The optical performances of the existing THz waveguides and sensors and the proposed sensor.

Ref.	Analyte	RS%	EMA	D(ps/	EML	NA
			(m ${}^{2}$ )	THz/cm)	(dB/cm)	
[5]	Cholesterol	98.75	-	±0.31	0.0008	0.35
	Blood					
[9]	Components	93.5	0.22	-	-	-
	Blood					
[10]	Components	99.39	7.7 × $10^{-8}$	±0.23	0.0014	0.38
	Human					
[13]	Body Protein	99.98	3.680 × $10^{-7}$	±0.006	3.33 × $10^{-5}$	0.08
	Blood					
[28]	Components	94.38	0.33	-	-	-
	Normal &					
[47]	Cancer Cell	92	1.25 × $10^{-8}$	±1.62	0.011	0.55
				0.15		
[48]	Chemical	95	9 × $10^{4}$	±0.15	0.012	0.50
PQF-RIBS	Normal and Cancer Cell	82.67	3.16 × $10^{-6}$	±0.35	2.53 × $10^{-9}$	0.99

## Data Availability

The data presented in this study are available upon request from the corresponding author.
